# Ocean warming and acidification affect the transitional C:N:P ratio and macromolecular accumulation in the harmful raphidophyte *Heterosigma akashiwo*

**DOI:** 10.1038/s42003-023-04524-8

**Published:** 2023-02-06

**Authors:** Satheeswaran Thangaraj, Jun Sun

**Affiliations:** 1 Institute for Advanced Marine Research, China University of Geosciences, Guangzhou, China; 2 State Key Laboratory of Biogeology and Environmental Geology, China University of Geosciences, Wuhan, China; 3 Department of Marine Science, Incheon National University, Incheon, South Korea; 4 Department of Physiology, Saveetha Dental College and Hospital, Saveetha Institute of Medical and Technical Sciences, Saveetha University, Chennai, India; 5 Research Centre for Indian Ocean Ecosystem, Tianjin University of Science and Technology, Tianjin, China

**Keywords:** Microbial biooceanography, Phenology

## Abstract

Despite an increase in ocean warming and acidification that is expected to increase the number of harmful algal species worldwide, the population of the raphidophyte *Heterosigma akashiwo* has been reported to be reduced. However, how this species physically and metabolically modifies transitional C:N:P ratio and macromolecule accumulation is unknown. Considering 1st, 10th, and 20th culture generations under present (low-temperature; low-carbon-dioxide [LTLC] 21 °C; *p*CO_2_ 400 ppm) and future (high-temperature; high-carbon-dioxide [HTHC] 25 °C; *p*CO_2_ 1000 ppm) ocean conditions, we examined transitional C:N:P ratio and macromolecule level changes and performed transcriptome sequencing. The results showed that compared to 1st generation cells, 20th generation cells under HTHC conditions showed a large decrease in carbon quota (Q_C_: 34%), nitrogen quota (Q_N_: 36%), and phosphorus quota (Q_P_: 32%), which were reflected in an overall reduction in DNA and RNA quantity. Decreased activation of photosynthetic, carbon fixation and lipid metabolic pathways coincided with changes in photosynthetic efficiency, carbon concentration, and lipid accumulation after long-term (20th generation) exposure to HTHC conditions. We observed that these variations in internal metabolic pathways were caused by external changes in temperature, which activated the (Ca^+^) signaling pathway, and external changes in *p*CO_2_, which altered proton exchange pathways. Our results suggest that *H. akashiwo* in a temperate environment will undergo profound changes in C:N:P ratio and macromolecular properties, leading to programmed cell death, in the future.

## Abstract (web summary)

Physiological experiments in *Heterosigma akashiwo* provide metabolic insights to the future of algal blooms under warming and acidifying ocean conditions.

## Introduction

Since the industrial revolution, the atmospheric carbon dioxide (CO_2_) concentration has nearly doubled, which has subsequently driven ocean warming and acidification. The excessive heat in the surface ocean due to increases in atmospheric temperature is predicted to increase by 3–5 °C, and the uptake of excessive CO_2_ is expected to increase *p*CO_2_ concentrations by 750–1000 ppm by the end of 2100^1^. These combined stresses may drive massive changes in phytoplankton ecosystems, as temperature and CO_2_ are directly correlated with cellular physiology^2^. A model study showed that the marine phytoplankton population may increase by as much as 40% under future *p*CO_2_ conditions^3^; however, the increase is expected to vary by species^4^. The frequency and severity of harmful algal blooms (HABs) in the past few decades have increased, partially due to ongoing climate change^5^. However, the species comprising HABs, such as raphidophytes, are being explored to understand their physiological changes^6^ caused by climate change.

The marine raphidophyte *Heterosigma akashiwo*, which is found worldwide^7^, produces ichthyotoxin, causing significant economic loss by killing fish^8^. Studies reported that light, temperature, nutrient levels, *p*CO_2_, salinity, and other microorganisms can alter *H. akashiwo* growth and bloom formation^9, 10^. For example, *H. akashiwo* blooms can also be triggered by excessive urea, ammonium, and nitrate, which are sources of nutrients^9^; for example, Wang et al. observed that this species grew well when exposed to various dissolved phosphates^11^. In the ocean environment, diel regulation of this species led to naturally increased photosynthetic and carbonic anhydrase (CA) gene expression with excessive uptake of phosphate to drive bloom formation^12^. However, other studies reported that the profound capability of a light-harvesting complex induced excessive assimilation of carbon and nitrogen, facilitating blooming^13^.

Elemental nutrients, described by the Redfield ratio (C:N:P)^14^, play key roles in phytoplankton growth and cell development^15^. Therefore, environmental factors that influence element storage are important to understand and predict phytoplankton survival under stress conditions^16^. Recent studies showed that temperature is a major factor altering the degree of element storage by phytoplankton^17^, while other studies observed that changing temperature alters stoichiometry and macromolecular allocation^18^. *H. akashiwo* under stress conditions reportedly exhibited decreases in the element ratio, gene expression related to macromolecules^12^, and nutrient acquisition^10^.

Temperature is a key factor affecting life forms on Earth, resulting in organisms developing signaling pathways and programs for regulating intracellular metabolism to optimize cell function^19^. The temperature or heat stress response (HSR) in *H. akashiwo* exposed to a temperature increase of 3–5 °C, underwent programmed cell death (PCD)^20^. However, no studies have examined how the combination of temperature and *p*CO_2_ changes drive PCD. In eukaryotes, protons play important roles in intracellular mechanisms, as all proteins rely on pH to maintain their shape and function, which are essential to metabolic processes^21^. Hence, changes in the extracellular pH may alter the intracellular pH and impact cytosolic components, secretory pathways, and the mitochondrial matrix with associated changes in cell physiology^22^. Recent reports noted that changes in the external pH in *H. akashiwo* cultures induced changes in swimming behavior^23^ and decreased element accumulation and gene expression in cells^24^. To date, several physiological and omics studies on *H. akashiwo* under different stress conditions have been reported. Nevertheless, no studies have examined how changes in the external environment, such as rising temperature and increased *p*CO_2_ concentrations, may influence or alter internal signaling processes to induce synergistic physiological changes.

Evaluating the synergetic impact of rising temperature and increased *p*CO_2_ often leads to questions about which factor contributes the most to changes in cellular function. However, as indicated by the Intergovernmental Panel on Climate Change^1^, the impacts of climate change (increased temperature and CO_2_) on phytoplankton are synergetic and should be studied together. It has also been suggested that the integrative effects between temperature and CO_2_ greatly increase the difficulty in performing multifactorial experiments to assess the impact of climate change^25^. Moreover, full factorial experiments that examine multiple variables at the same time are operationally impractical^26^. Therefore, Boyd et al.^26^ proposed incorporating multiple factors into “clusters” to enable experimentation that can provide insights into the interactive impacts of two or more variables. Thus, multiple environmental factors under the present circumstances can be combined into a single cluster and compared to a “future cluster” of variables based on the IPCC scenario.

In this study, we examined the impact of the combination of rising temperature (4 °C) and increasing *p*CO_2_ (1000 ppm) on *H. akashiwo* using physiological measurements and transcriptome analysis to understand the intracellular signaling processes related to element (C:N:P) and macromolecule accumulation. Thus, we assessed (i) intermediate variations in element and macromolecule accumulation after prolonged exposure to a combination of increased temperature and *p*CO_2_, (ii) prolonged exposure-induced changes in the expression of genes related to growth and cell development, and (iii) the mechanisms by which increased external temperature and *p*CO_2_ in culture medium drive intracellular metabolic modifications. To investigate these areas of interest, *H. akashiwo* was cultured through 20 generations. Then, we performed diverse physiological measurements of the 1st, 10th^,^ and 20th *H. akashiwo* generations and compared the findings. In addition, a transcriptome analysis was performed with the 20th generation cells under LTLC and HTHC conditions to understand the metabolic variations induced by the physiological changes caused by prolonged exposure to climate change conditions.

## Results

### Cellular physiology after temperature and *p*CO_2_ were increased

**Fig. 1:**
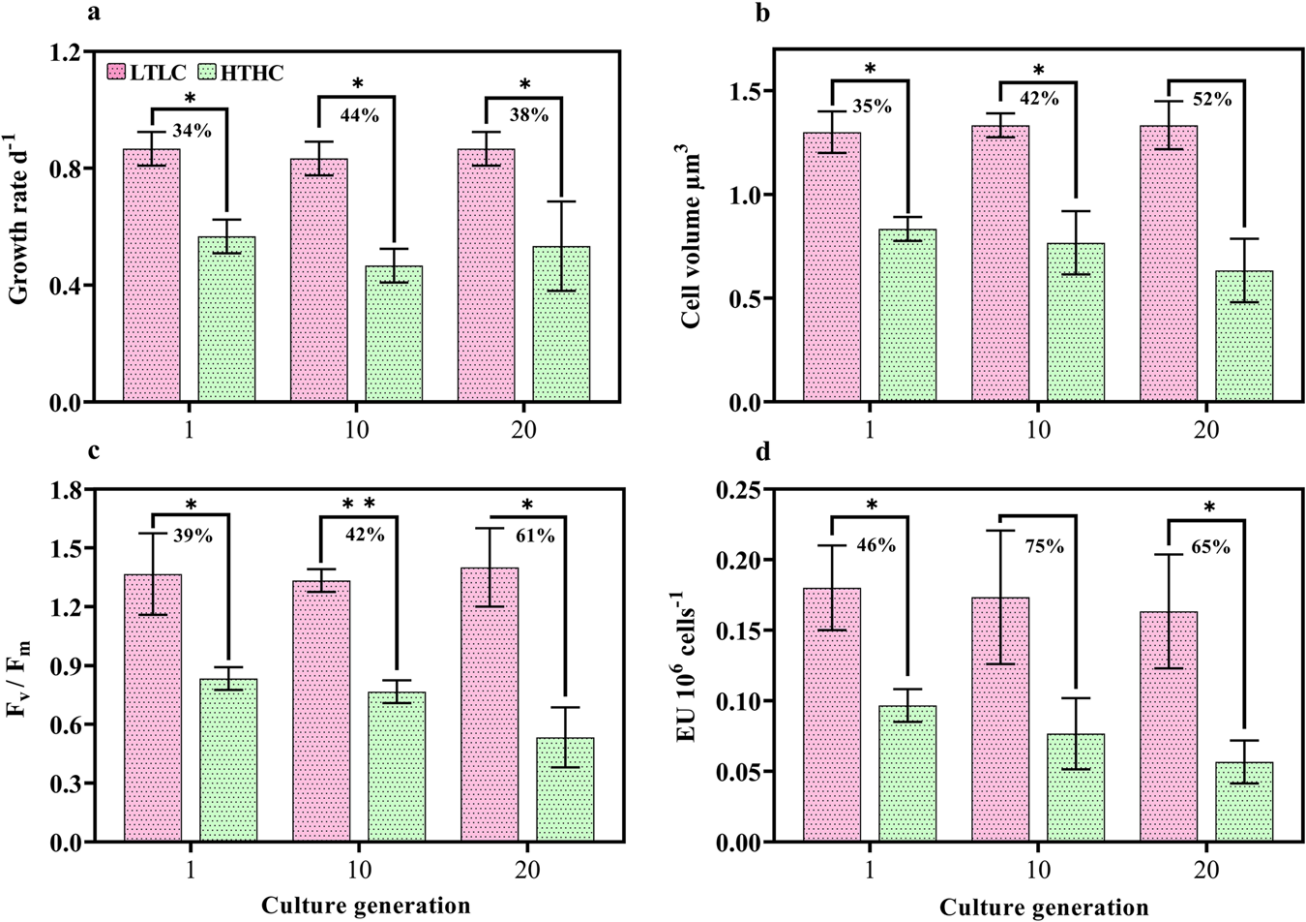
Physiological response of *Heterosigma akashiwo* grown to different generations under LTLC and HTHC conditions. **a** Specific growth rate (**b**) Cell volume, (**c**) photochemical yield, and (**d**) carbonic anhydrase activity. Vertical bars in each graph represent triplicate samples (SD, *n* = 3). The percentage indicates comparison values between the LTLC and HTHC samples. Significant differences for each parameter relative to the control are denoted by ^∗^*p* < 0.05 and ^∗∗^*p* < 0.001.

The cellular physiology of different generations of *H. akashiwo* cultured under LTLC and HTHC conditions is shown in Fig. 1. The average growth rate (Fig. 1a) of *H. akashiwo* cultured under HTHC conditions (0.52 d^−1^) was 38% lower than those cultured under LTLC conditions (0.85 d^−1^); HTHC cells of the 10th and 20th generations showed a similar decrease in growth rate compared to cells in the LTLC environment (both *p* < 0.05). However, the volume of the HTHC cells in the 20th generation showed a greater reduction than that of the cells in the 10th generation (Fig. 1b). In addition, compared to the LTLC treatment, the HTHC treatment led to lower mean levels of photosynthetic compounds (F_v_/F_m_) (47%, Fig. 1c [all *p* < 0.05]) and CA (66%, Fig. 1d).

**Fig. 2:**
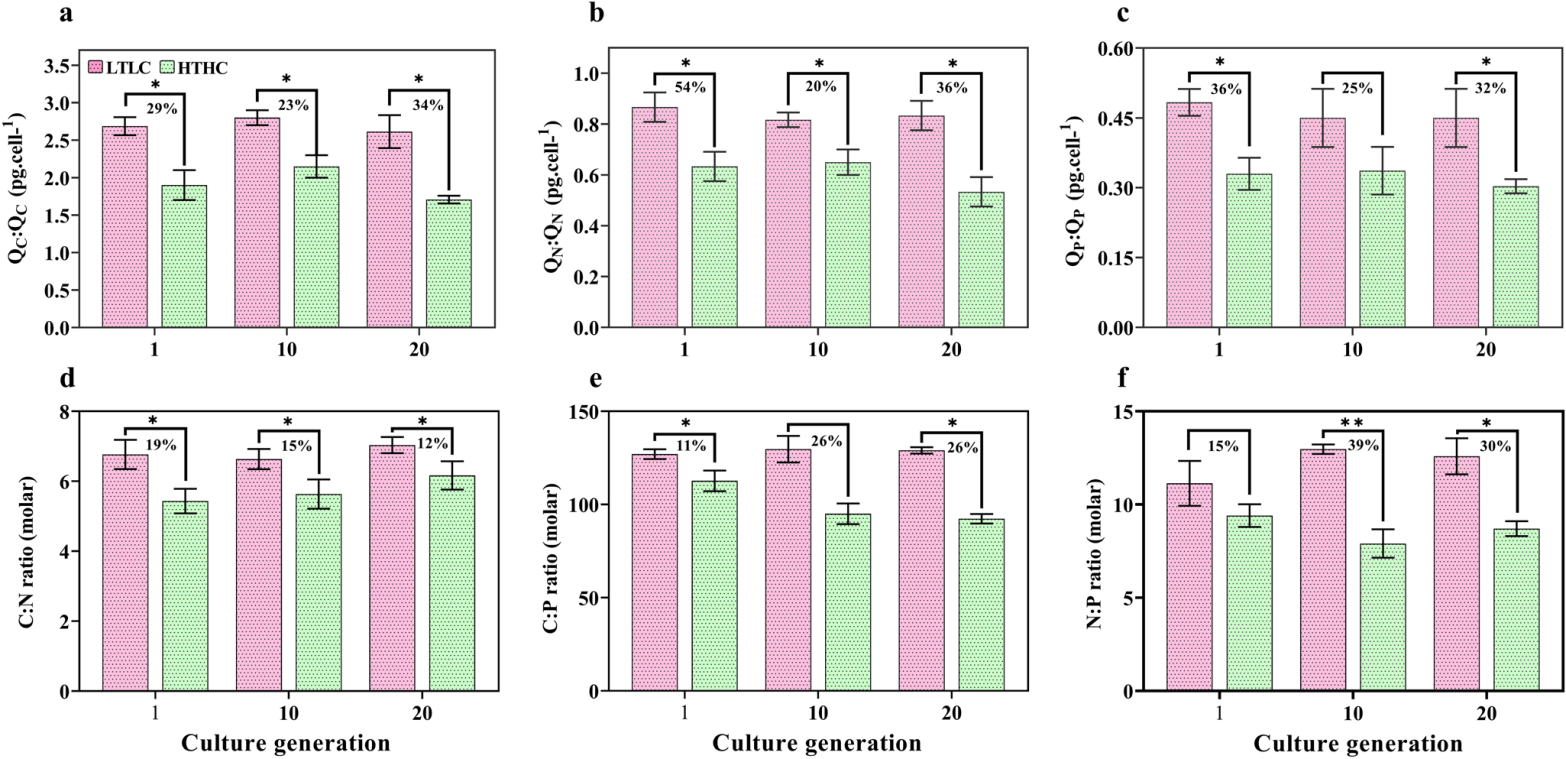
Elements and stoichiometry changes in *Heterosigma akashiwo* grown to different generations under LTLC and HTHC conditions. **a** carbon (**b**) nitrogen, (**c**) phosphorus quotas, and (**d**) cellular carbon:nitrogen, (**e**) cellular carbon:phosphorus, and (**f**) cellular nitrogen:phosphorus stoichiometry. The percentages indicate comparison values between the LTLC and HTHC samples. Significant differences for each parameter relative to the control are denoted by ^∗^*p* < 0.05 and ^∗∗^*p* < 0.001.

There was a substantial reduction in the C:N:P quotas of HTHC cells compared with LTLC cells in all generations (Fig. 2a–c). However, among the HTHC cells, a slight increase in element accumulation was noticed in the 10th generation cells, but in the 20th generation cells, the levels of all the elements declined further. At the end of the experiment, compared to C and P contents, the HTHC cell N content was reduced the most (36%, *p* < 0.05), and all the element levels were reduced to a greater extent in compare to the LTLC cells. Despite a decreasing trend in *H. akashiwo* C accumulation (Fig. 1d), the C:N ratio differed (Fig. 2d; all *p* < 0.05), showing an increasing trend (up to 13%) between the 1st and 10th generations of HTHC cells. In contrast, the C:P ratio in HTHC cells showed a gradual decline (11–26%) compared with that in the LTLC and between the different generations of HTHC cells (Fig. 2e). In contrast to the C:N ratio, the C:P and N:P ratios in the HTHC cells showed a twofold reduction in the 20th generation compared to the first generation (Fig. 2e–f). However, similar to other stoichiometries, the N:P ratio in HTHC cells was found to be reduced throughout the experiment (15–30%) compared to that in the LTLC cells.

**Fig. 3:**
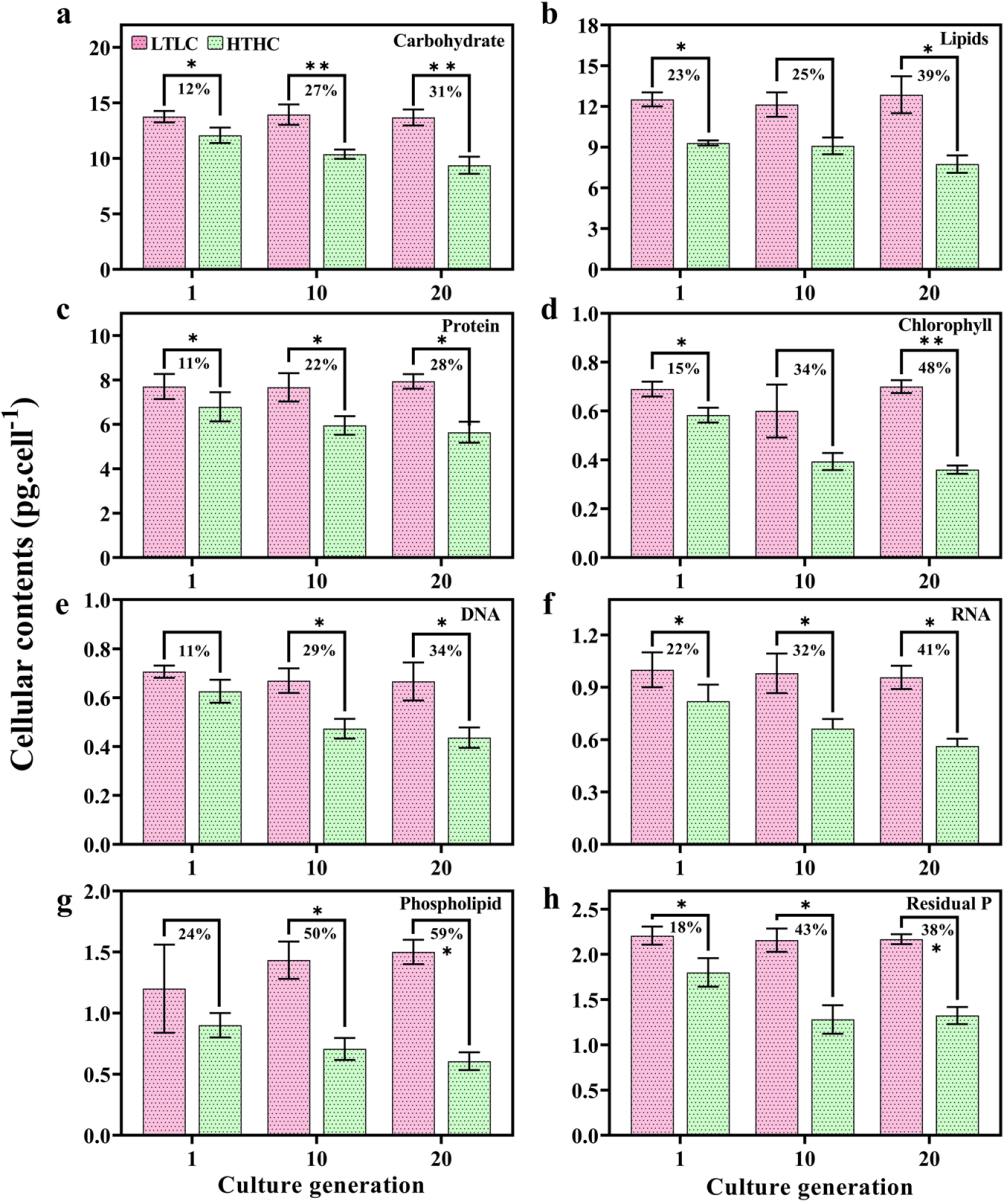
Variation in the macro (carbohydrate, lipid, and protein) and minor (chlorophyll, DNA, RNA, phospholipid, and residual phosphorus) molecular content in *Heterosigma akashiwo* grown to different generations under LTLC and HTHC conditions. **a** carbohydrate (**b**) lipids, (**c**) protein (**d**) chlorophyll (**e**) DNA (**f**) RNA (**g**) phospholipid and (**h**) residual P. The percentages indicate comparison values between LTLC and HTHC samples. Significant differences for each parameter relative to the control are denoted by ^∗^*p* < 0.05 and ^∗∗^*p* < 0.001.

The macromolecular contents identified in different generations are shown in Fig. 3a–h. At a glance, all the contents in the HTHC cells seemed to show a decreasing trend; however, some of these macromolecule levels were differentially changed in different generations. Specifically, compare to LTLC cells carbohydrates showed the largest decreases between the 1st and 10th generations of HTHC cells (12–27%; both *p* < 0.05), whereas a smaller reduction (27–31%; both *p* < 0.001) was observed between the 10th and 20th generations of HTHC cells (Fig. 3a). Lipid accumulation showed the opposite trend, with smaller decreases (23–25%) between the 1st and 10th cell generations and larger decreases (25–39%) between the 10th and 20th cell generations (Fig. 3b). The declining trend in protein levels in HTHC cells remained similar throughout the experiment with significantly (*p* < 0.05), representing an 11% (between the 1st and 10th generations) and a 22% (between the 10th and 20th generations) reduction compared to the decreased protein levels in LTLC cells (Fig. 3c). The chlorophyll content among HTHC cells showed a rapid decline between the 1st and 10th cell generations (32%; *p* < 0.002) and the 10th and 20th cell generations (8%) and was nearly twofold (38%; *p* < 0.0003) that of the 1st cell generation (Fig. 3d). Both the DNA and RNA levels in HTHC cells showed a similar declining trend compared to that in LTLC cells, with the former showing an 11–34% decrease in DNA and a 22–41% (all *p* < 0.05), decrease in RNA (Fig. 3e–f). This trend suggests that compared to the 1st cell generation, the DNA and RNA contents decreased nearly twofold in the 20th generation, showing that prolonged exposure to changing climate conditions severely impacted these nucleotides. Although phospholipid and residual P levels were associated with cellular P levels, they showed different responses after long-term exposure to changing climate conditions (Fig. 3g–h). For example, both phospholipid and residual P levels among HTHC cells showed a similar level of decline between the 1st and 10th generations (a 21% decline in phospholipids and a 32% decline in residual P). However, between the 10th and 20th generations, the phospholipid level decreased further, by 14%, but the residual P level increased by 2%. This variation suggests that with prolonged exposure to HTHC conditions, the residual P level in *H. akashiwo* increased, possibly to support acclimation.

### Changes in photosynthesis and related functional activities

The de novo sequence assembly led to the identification of 126555 genes in *H. akashiwo* in this study, and nearly 50% of these genes were annotated in different databases (Table S1, Supplementary material). Among these identified transcripts, 18421 differentially expressed transcripts/genes (DET/DEGs) were observed, and the levels of 14730 (74%) of these DETs/DEGs were decreased, and those of 3691 (26%) DETs/DEGs were increased (Supplementary data 2). The enriched terms metabolism of photosynthesis, light-harvesting complex (LHC), chlorophyll biosynthesis, and oxidative phosphorylation indicated the metabolic pathways that were influenced by the HTHC conditions in this study (Supplementary data 3). In the photosynthetic pathway, 56 DEGs were identified, and 17 of these DEGs were upregulated, including *PsbU*, the gene encoding oxygen-evolving enhancer protein, and *Psb27*, whereas 39 of the DEGs were downregulated, including those that encoded several F-type H^+^-transporting ATPase subunits. In total, 45 DEGs were detected in the LHC, 24 of these transcripts were upregulated, and 21 of these transcripts were downregulated. These genome changes in LHC genes subsequently altered chlorophyll biosynthesis metabolism, for which 10 transcripts were upregulated, and 25 transcripts were downregulated. Furthermore, 155 transcripts related to ATP metabolism were differentially expressed, with only 8 transcripts upregulated and 147 downregulated. Transcripts related to ATP synthase, proton transport, quinone binding, and rotational mechanisms were the notably downregulated transcripts.

### Functional pathways associated with carbon and lipid metabolism

Similar to photosynthetic physiology pathways, the glycolytic, citrate, pyruvate, and carbon fixation metabolic pathways, which were related to carbon and carbohydrate accumulation, in *H. akashiwo* were altered after prolonged exposure to HTHC conditions (Supplementary data 4). Glycolysis is the primary mechanism through which glucose is converted into pyruvate. Of these 127 DEGs in glycolysis, 15 genes were upregulated, and 112 genes were downregulated. The levels of five *glyceraldehyde-3-P dehydrogenases* (GADPH) critical for the conversion of *glyceraldehyde 3-phosphate* to *D-glycerate 1,3-bisphosphate* were decreased, by 1.43–4.73 fold. The level of the enzyme *pyruvate kinase* (PK) was reduced by 3.58–3.46 fold during the transfer of a phosphate group from phosphoenolpyruvate to adenosine diphosphate during the synthesis of pyruvate molecules. The mRNA abundance of *hydroxymethyl glutathione* was decreased by 5.12–5.97 fold. *Hydroxymethyl glutathione*, in collaboration with *L-lactate dehydrogenase* (D-LDH), transforms methylglyoxal into pyruvate, which directly enters the citrate cycle. Four *L-lactate dehydrogenases* were detected, and their expression was downregulated by 3.61–5.03 fold. These results suggest that long-term exposure to HTHC conditions reduced the level of byproducts generated during the glycolytic production of pyruvate in *H. akashiwo*.

In total, 126 transcripts associated with the TCA cycle were differentially expressed, and the expression of 120 of these genes was downregulated, and that of 6 genes was upregulated. In total, 15 transcripts encoding *isocitrate dehydrogenase* (IDH) were downregulated by 1.15–8.89 fold. IDH catalyzes oxidative decarboxylation and produces 2-oxoglutaric acid (2-OG), which is a precursor in the absorption of ammonium. The expression of three *fumarate hydrates* was decreased by 1.37–3.84 fold. The expression of three *malate dehydrogenases* (MD) was decreased by 1.93–5.28 fold, and one MD, which catalyzes the reversible oxidation of malate to oxaloacetate, was increased by 1.3-fold. In addition, 9 *citrate synthases* (CSs) were differentially expressed, with all showing a decrease of 1.72–7.71 fold. CSs catalyzes the condensation of acetyl coenzyme A with oxaloacetate to generate six-carbon citrate, which is the first step in the TCA cycle. Furthermore, 143 DETs involved in pyruvate metabolism were identified, with the levels of 17 DETs increased and those of the remaining 126 decreased. Therefore, with decreased photosynthesis and LHC, carbon fixation metabolism was characterized by 75 DEGs, with 54 downregulated and 21 upregulated. This metabolic variance was consistent with our physiological measurements of CA activity (Fig. 1d), which showed a similar pattern of decreased carbon accumulation in HTHC cells compared with LTLC cells.

A total of 88 DEGs involved in fatty acid biosynthesis metabolism were identified, 14 were upregulated and 74 were profoundly downregulated (Supplementary data 5). Of these DEGs, one encoding an important *2-long-chain acyl-CoA synthase* was upregulated by 2.13–13 fold, whereas 19 DEGs were downregulated by 1.79–10.07 fold. Similarly, 94 DETs involved in fatty acid degradation were identified, with levels of 4 increased and 90 decreased, including transcripts encoding the important enzymes *enoyl-CoA hydratase* and *methylglutaconyl-CoA hydratase*. The fatty acid elongation pathway comprised 21 DEGs, with 9 upregulated and 12 downregulated. Six DEGs were identified in the ether lipid metabolism pathway, and four of these DEGs were downregulated and two were upregulated. Taken together, in both carbon and lipid metabolism pathways, the levels of most of the DETs critical for carbon and lipid accumulation were increased, which may have reflected the lower carbohydrate and lipid levels in HTHC cells (Fig. 3a–b).

### Variations in DNA and RNA biosynthesis mechanisms

Five different metabolic pathways related to DNA biosynthesis were profoundly altered: The DNA replication and nucleotide, base, and mismatch repair mechanism pathways (Supplementary data 6). Among these pathways, the nucleotide repair metabolism pathway showed the greatest alteration, affecting 62 DETs (with the levels of 49 transcripts decreased and those of 13 increased), followed by the base repair mechanism pathway (32 DETs, with the levels of 25 decreased and those of 7 increased), and mismatch repair mechanism pathway (26 DEGs, with 20 downregulated and 6 upregulated). Similarly, the DNA replication and homologous recombination pathways involved 25 and 24 DEGs, of which 52% were involved in DNA replication and 85% involved in homologous recombination showed decreased expression.

Similar to the DNA pathways, five metabolic pathways were associated with RNA biosynthesis: RNA degradation, polymerase, transport, mRNA surveillance, and spliceosome pathways (Supplementary data 7). In the RNA degradation pathway, 79 DEGs were identified, with the levels of 64 transcripts reduced and those of 15 transcripts increased. Among the other 9 molecular chaperones, the expression of genes related to enzyme-mediated folding of polypeptides was downregulated by 1.28–4.73 fold, with none upregulated. Furthermore, of the 186 DETs related to RNA polymerase identified, with the levels of 157 DETs decreased and those of 29 DETs increased. This large decline in the levels of RNA polymerase-related transcripts may have impacted the transcription of DNA sequences into RNA. In the mRNA life cycle (RNA transport) metabolism pathway, 939 DEGs were identified; of these DEGs, the levels of 153 transcripts were increased, and those of 786 transcripts were decreased. Similarly, 694 DETs in the mRNA surveillance pathway were identified, of which the levels of 109 transcripts were increased, and those of 585 transcripts were decreased. The largest RNA‒protein complex in the spliceosome was associated with 273 DETs, with the levels of 47 transcripts increased and the levels of the remaining 226 transcripts decreased.

### Mechanistic regulation of protein biosynthesis

Six metabolic pathways related to protein biosynthesis were modified under HTHC conditions (Supplementary data 8). Of the enzymes in these pathways, aminoacyl-tRNA synthases played central roles in the translation of 81 DETs, with the levels of 77 decreased and those of 4 increased. The reduced transcript levels included those of several *leucyl-tRNA* synthetases (which catalyze covalent ligation), *lysyl-tRNA* synthetases (which form lysyl-transfer RNA), and *isoleucyl-tRNA* synthetases (which aminoacylate tRNA with isoleucine). Ribosome biogenesis, critical for the production of ribosomes, was associated with 111 DETs, with the levels of 56 transcripts increased and those of 55 transcripts decreased. The transcripts that were increased included those encoding smaller peptides with nucleolar functions (such as the snoRNA-binding domain) and CCT motifs, while the transcripts that underwent level reduction encoded nucleolar proteins and adenylate kinases. These changes in ribosome biogenesis were reflected in 352 DETs associated with ribosome metabolism, of which the levels of 95 transcripts were increased, and those of 257 transcripts were decreased, including those encoding large- and small-scale subunits. Metabolism in the endoplasmic reticulum (ER) (which produces proteins in the cell) was associated with 175 DEGs, of which 153 transcript levels were decreased and 22 transcript levels were profoundly increased. Several genes involved in major protein degradation pathways, such as the proteasome and endocytosis pathways, were differentially expressed under HTHC conditions. Of these DEGs, endocytosis (critical for lysosomal proteolysis) was associated with 260 DETs, with the levels of most (223) transcripts decreased and those of 37 transcripts increased. Proteasome metabolism (critical for the degradation of proteins) was associated with only 10 DETs, with the levels of 6 transcripts decreased and those of 4 transcripts increased.

## Discussion

Here, we examine the physiological and transcriptome responses of *H. akashiwo* related to elemental (C:N:P), stoichiometric, and macromolecular changes to future ocean conditions. The levels of all the analyzed elements and macromolecule accumulation and the respective metabolic rates decreased under HTHC conditions. Specifically, HTHC cells showed lower nucleotide (DNA and RNA) and protein contents with downregulated activation of nucleotide and protein biosynthesis pathways. This metabolic decline may be the cause of the reduced transcription efficiency and growth rate in species acclimating to climate change^27^. Moreover, these results suggest that the influence of transcription on the translation mechanisms in *H. akashiwo* under future ocean conditions may not be a simple function of changes to resource allocation strategies. Hennon et al.^24^ showed increased cellular elements in *H. akashiwo* with a *p*CO_2_ ranging from 700 to 800 ppm and demonstrated a trend of decreasing element levels and related metabolic activity when *p*CO_2_ exceeded 800 ppm. Similarly, elements and metabolic pathways in this study showed decreasing patterns at 1000 ppm *p*CO_2_. Notably, the stoichiometry of *H. akashiwo* generally varied in different growth phases because dynamic allocation and nutrient demands tended to change based on the surrounding environment^28^. Cell absorption of surrounding nutrients results in intracellular accumulation and, to a lesser degree absorption. In this study, we did not analyze intracellular accumulation directly, and this should be a focus of future works analyzing climate change conditions. In phytoplankton, a higher contribution of cellular Q_N_ is consumed for protein functions, and cellular Q_P_ is consumed for ribosomal RNA functions^29^. Therefore, elemental stoichiometry, such as N:P, is determined by the protein:rRNA ratio, reflecting the physiological requirements^30^ altered by the surrounding environment. Therefore, similar trends of Q_N_ and Q_P_ decline in this study indicated a similar impact on the N:P ratio in HTHC cells.

*H. akashiwo* engages specified photoprotective mechanisms to overcome excessive solar radiation^31^, and LHC degradation changes to adapt to changes in nutrient levels, supporting other metabolic processes^32^. We identified that the levels of several photosynthesis-related transcripts were reduced (39 of all 56 DETs), but the levels of a high proportion of DETs in the LHC were increased (24 of all 45 DETs), and these DETs were related to light absorption and dissipation. Carbon obtained from gene upregulation in the LHC tended to preserve the metabolic integrity of the cell by protecting the photosynthesis apparatus and maintaining a balanced cellular C:N ratio^33^. In addition, a high number of genes expressed in the LHC in *H. akashiwo* were considered to show genetic advantages, which led to the dominating presence *H. akashiwo* among the species in the natural environment^13^. It has been reported that changes in the *H. akashiwo* chloroplast mechanism are reasons for its adaptation when cells are exposed to high-light conditions^34^. In this study, we observed a reduction in HTHC chlorophyll content but profound increases in the levels of transcripts involved in LHC metabolism related to the maximum light acquisition. Despite decreasing trends in growth and element levels after prolonged exposure to HTHC conditions, *H. akashiwo* showed the ability to manage the LHC, possibly in response to changing environmental conditions.

The decreased expression of core carbon metabolic pathway components suggested a reduction in the efficiency of biophysical carbon-concentrating mechanisms (CCMs), leading to a slower growth rate. Studies have described a reduction in the levels of CCM transcripts in phytoplankton, as measured through ATP levels and carbon skeletons, which are essential for N assimilation and degradation^35^. The decreased metabolism observed in this study may underpin changes in the C:N and N:P ratios and related macromolecule levels in *H. akashiwo* in the future ocean. Carbonic anhydrases (CAs) in CCMs facilitate intra- and extracellular conversion between HCO_3_^−^ and CO_2_. A high proportion of intracellular CAs, such as 15 *α*-CA, three *β*-CAs, and three *γ*-sCA, were differentially expressed in this study (Supplementary data 2). Of the 15 α-CA transcripts identified, the levels of 13 transcripts were decreased, by 0.85–8.12 fold, with an average decrease of 2.49 fold. Of the three *β*-CAs transcripts, the levels of all were reduced by an average fold change of 4.79, and of the three γ-CA units, the levels of two transcripts were decreased, and the level of one transcript was increased. Decreases in CA activity were consistent with our physiological CA measurements (Fig. 1d). In coastal environments, increased CA activity in *H. akashiwo* has been reported to increase CCM activity and thus increase cellular growth and the replication rate, causing bloom formation^13^. Increased activity of these CAs in the diatom *Skeletonema dohrnii* led to increased carbon accumulation with subsequent carbon allocation to lipids and nucleotides, which enhanced the growth and proliferation of this species under HTHC conditions^36^. In addition, eleven solute carrier family transcripts (SLC) critical for transmembrane bicarbonate transport were identified in the present study (Supplementary data 2). Of these transcripts, the levels of 9 transcripts have reduced an average of 1.88-fold, and the levels of two of these transcripts were increased, by 1.86-fold. A substantial contribution of SLC transcripts to carbon metabolism and increased growth potential under varying CO_2_ conditions in diatoms has been documented^37^. Under blooming conditions, the rapid consumption of CO_2_ by high biomass of phytoplankton reduces the available CO_2_ level;^38^ therefore, *H. akashiwo* expresses an HCO_3_^−^ transport system and increases the abundance of CAs to acquire sufficient CO_2_ to support their rapid proliferation. We speculate that with decreased CA and SLC transcript levels and prolonged exposure to HTHC, the inorganic carbon transportation and fixing capabilities of *H. akashiwo* were reduced to support their effective growth and proliferation. Considering these collective data, we speculate that the combination of increased temperature and *p*CO_2_ in the future may trigger profound changes in the elements (C:N:P) available to the Heterosigma community and their stoichiometry within species, causing macromolecular metabolism shifts that lead to slower growth and proliferation.

**Fig. 4:**
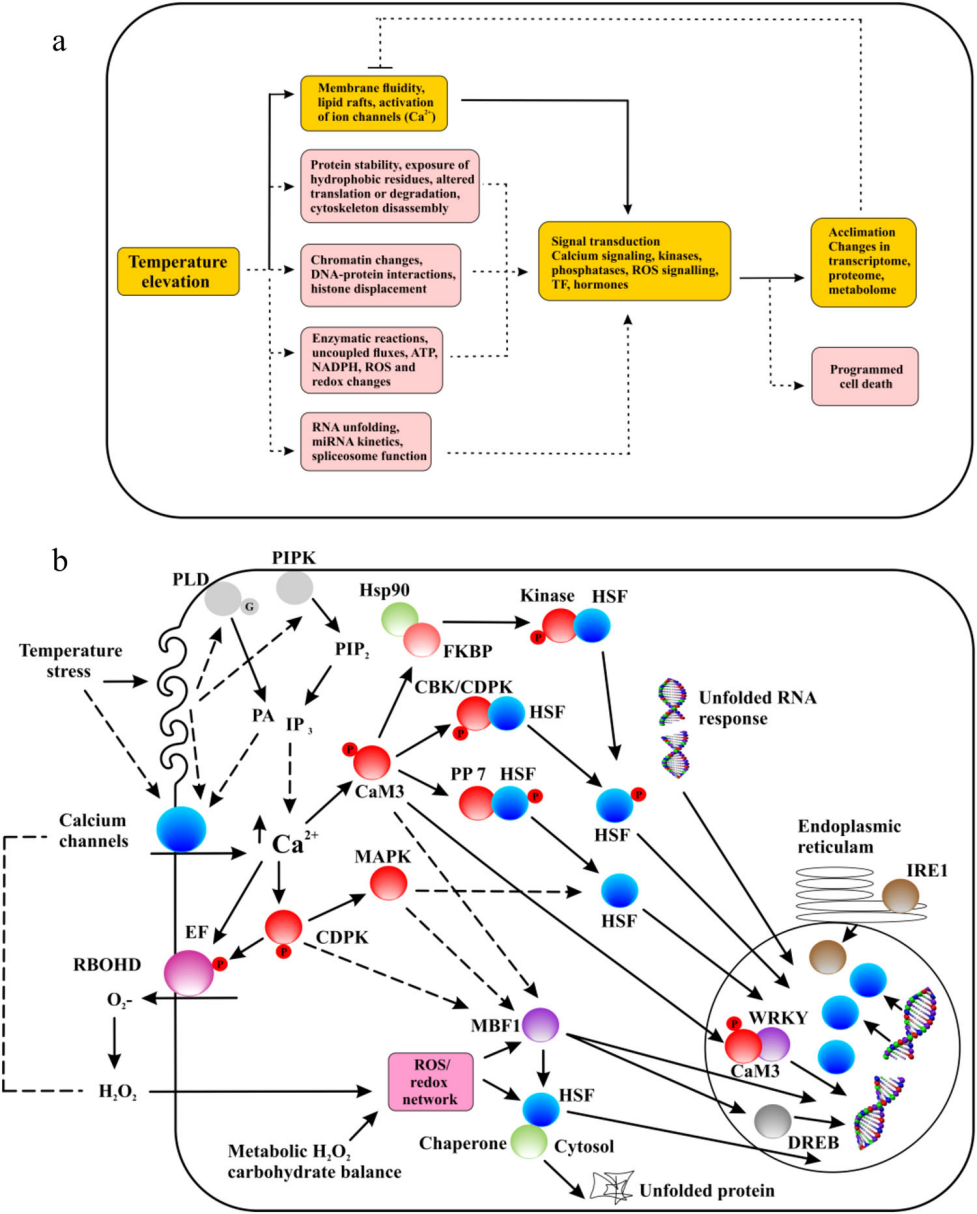
Temperature-sensing scheme in algae. **a** Changes in temperature impact diverse processes in algae. Even a small change in temperature can alter algal membrane properties and subsequently activate calcium channels. The influx of calcium activates signal transduction and regulates intracellular metabolism in an acclimation process. The highlighted yellow pathways and linked solid arrows indicate primary temperature sensing mechanisms in algae and plants, respectively. Temperature changes induce further modification in protein stability, which may trigger the unfolded protein response (UPR) in the cytosol and endoplasmic reticulum (ER), ROS accumulation, changes in cellular energy levels, and —unfolding of RNA, which might act as riboswitches and/or impact spliceosome- and microRNA-influenced metabolic functions. Pathways (boxes) shown in light pink are thought to activate signal transduction changes that help cells to acclimate. Furthermore, an alternative acclimation mechanism involves the activation of programmed cell death (PCD), which can be induced by a combination of different signal transduction conditions or pathways. **b** A schematic model showing the signal transduction pathway activated in response to temperature stress in algae. Temperature stress impacts membrane stability and promotes the activation of plasma membrane calcium channels, resulting in inward flux of calcium (left side). This membrane stability change may also trigger lipid signaling via the lipid-modifying enzymes phospholipase D (PLD) and phosphatidylinositol-4-phosphate 5-kinase (PPIK). Calcium binds CaM3 (calmodulin) and triggers the transcriptional regulators of the heat stress response (HSR), i.e., heat shock transcription factor (HSR), MBF1c, dehydration-responsive element-binding (DREB), and WRKY. Elevation of calcium in the cytosol also activates the ROS-producing enzyme RBOHD (respiratory burst oxidase homolog D) located in the plasma membrane via direct interaction or through the activation of calcium-dependent protein kinases (CDPKs), which phosphorylate RbohD (*lower left*). ROS derived from RbohD, such as superoxide (O_2_^−^), which is converted to hydrogen peroxide (H_2_O_2_), can cause membrane depolarization and enter cells to activate ROS production and/or the redox signaling network, which in turn activate HSFs and MBF1c. During temperature stress in cells, ROS may accumulate due to metabolic imbalances and activate the same networks. Two unfolded protein response (UPR) pathways are shown; one is located in the ER (ER-UPR) and the other is located in the cytosol (Cyt-UPR). The Cyt-UPR functions through HSFs, while the ER-UPR functions through a sensor molecule (IRE1) and the transcription factors bZIP60, bZIP2817, and bZIP17. In the nucleus, temperature elevation decreases the H2A.Z (occupancy of histone) at HSP promotors (*right side*).

Gene expression can be regulated by external factors derived from the organism’s living environment and/or internal factors that originate within the organism^39^. Through internal metabolism, cells communicate by releasing or receiving energy and inducing signaling cascades that lead to the transcription of genes by turning genes on or off or by modifying the transcripts produced, leading to changes in cell behavior and morphology^39^. Among external factors, temperature variation has been reported to be a strong influence on the gene expression profile that leads to subsequent changes in cell physiology^40^. In algae, temperature stress (TS) is involved in multiple pathways triggered by four sensors: a plasma membrane (PM) channel (which initiates inward calcium flux), a histone sensor in the nucleus, and two unfolded protein sensors in the cytosol and ER. The intracellular metabolic variations induced by TS can alter membrane stability and RNA and protein structures, leading to metabolic imbalance and reactive oxygen species (ROS) accumulation^41^. To prevent excessive ROS production, algae and plants undergo lipidome, transcriptome, and proteome expression reprogramming to optimize their internal temperature (Fig. 4a). These metabolic alterations establish a new metabolic steady state that allows organisms to function, survive and reproduce at higher temperatures.

When algae are exposed to high temperature, their surface ensures that all macromolecules perceive the stress simultaneously. These macromolecules induce physiological changes, i.e., increased membrane fluidity, a partial reduction in DNA and RNA levels, protein subunit disassociation, and related changes to their hydrophobic core (Fig. 4a). Thus, in theory, all macromolecules act as thermosensors by delivering output in the based on the degree of functional loss^42^. Hence, we assume that TS (induced via elevated temperature) in this study could be played a primary role in decreasing the levels of macromolecules and related metabolic pathways. To activate the heat sensor molecule in algae, metabolic pathways eliminate ATP from cells and reduces energy levels (metabolic flux). During this process, TS-induced protein unfolding relieves chaperone inhibition by enabling them to dissociate from their inhibitory partners and subsequently bind and activate unfolded proteins^42^. The primary event leading to TS sensing is frequently realized at the PM^42, 43^. A molecular investigation has revealed that even small temperature variations are detected at the PM, which triggers the influx of calcium into the cell through specific channels to activate the HSR^43^.

TS induces calcium influx (Fig. 4b), which regulates several signaling pathways. In plants, calmodulin CaM3 requires TS signaling^44^ and is involved in the activation of different transcription factors (TFs), such as the heat shock transcription factor (HSF) and WRKY39 transcription factor^45^. This calcium influx activates diverse calcium-dependent protein kinases (CDPK), which then trigger the ROS-producing NADPH oxidase enzyme^41^ or mitogen protein kinases (MAPKs)^46^. CaM3 also activates calcium-/calmodulin-binding protein kinase, which phosphorylates HSFA1a (an HSP component), whereas phosphatase-007 dephosphorylates HSFA1^47^. The HSP90/FKBP kinase (ROF1, also known as FKBP62) can mediate HSF phosphorylation caused by calcium-binding calmodulin^48^. Furthermore, to activate PM ion channels, temperature-induced membrane fluidity changes lipid signaling. Thus, TS results in the activation of *phosphatidylinositol-4-phosphate 5-kinase* (PIPK), *phospholipase D* (PLD), and other lipid signaling molecules that accumulate, including phosphatidic acid, D-myo-inositol-1,4,5-trisphosphate (IP_3_) and phosphatidylinositol-4,5-bisphosphate (PIP_2_)^49^. Furthermore, decreased phospholipase C9 activity has been directly related to decreased IP_3_ concentration, decreased HSF, and decreased heat tolerance^50^. The buildup of lipoid signaling molecules results in the opening of channels and calcium influx.

The unfolded protein response (UPR) pathway is activated in cells in response to stress that impairs protein stability in the ER^51^. There are two UPR pathways located: one is located in the cytosol, and the other is located in the ER^52^. Different metabolic pathways likely rely on enzymes with varying heat sensitivity. It has been proposed that TS may uncouple certain metabolic processes and induce the production of ROS, which might function as signals to activate the HSR^53^. Because ROS buildup at the PM outer surface can result in membrane depolarization^48^_,_ calcium-induced ROS-producing enzyme (RBOHD) activation and ROS accumulation may exacerbate the HSR by activating additional calcium channels in the PM (Fig. 4b). Furthermore, ROS buildup in cells can cause PCD^41^, explaining the cause of HSR-induced PCD (Fig. 4a–b). In general, we hypothesize that the decrease in growth rate, macromolecule content, and related gene expression/metabolic modification shown in the HTHC cells in this study may be a result of signaling generated by different sensors integrated into signal transduction networks in response to TS.

**Fig. 5:**
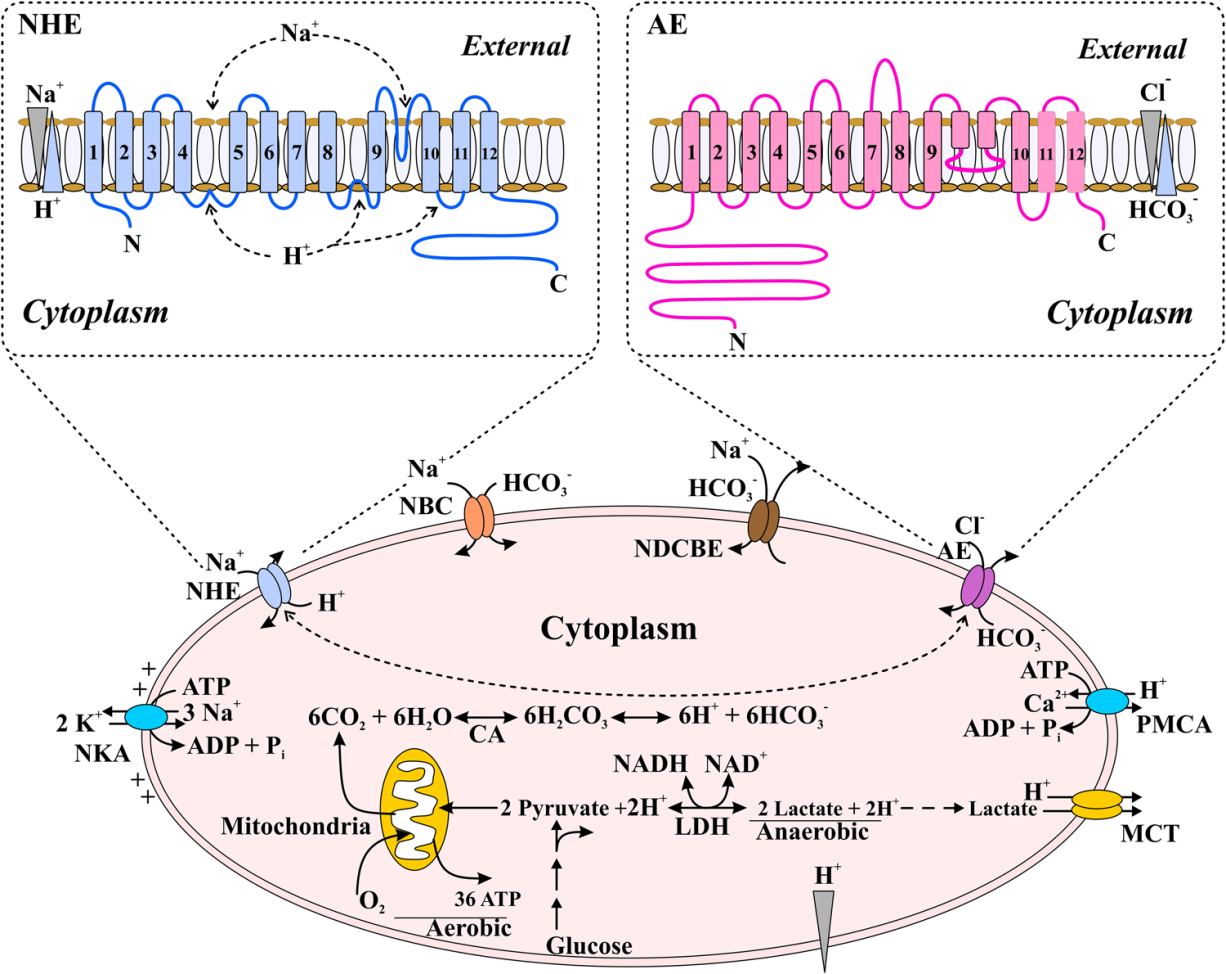
The regulation of cytoplasmic pH by ion carriers in algal metabolism. The cytoplasm tends to be acidified via various metabolic activities/pathways, i.e., oxidative phosphorylation in mitochondria that produces CO_2_ (the aerobic component in the figure) and ATP production by glycolysis that generates lactate (the anaerobic component in the figure). pH transporters (Na^+^ –HCO_3_^−^ cotransporters [NBCs] and Na^+^–H^+^ exchangers [NHEs]) are critical for plasma membrane alkalinization. These transporters consume energy that has been stored in electrochemical Na^+^ gradient, which is established by (NKA) Na^+^ –K^+^ ATPase pumps to drive solute transport. These (alkaline) mechanisms are balanced by the activities of PM Cl^−^– HCO_3_^−^/anion exchangers (AEs), which acidify the cell. PM Ca^2+^ ATPases (PMCAs), which exchange H^+^ in the cytosol with extracellular H^+^, further acidifying cytosolic components to elevate the intracellular Ca^2+^ level. In cells, alkalinization may be favored by MCT monocarboxylate H + cotransporters. The NHE and AE proteins are shown in the insets, and carbonic anhydrase (CA), lactate dehydrogenase (LDH), cytosol pH, pH_c_, pH_o_, and extracellular (or outside) pH are shown.

In eukaryotes, protons dictate macromolecule structure and function, which can be used as energy currency^22^. Therefore, unique organelle activity is dependent on the preservation of a specific pH. The consumption or production of proton equivalents depends on the prevailing pH and the ability of a cell to respond to variations in transport pathways. A balance between proton import and export processes underpins pH stability not only in the cytosol but also in the ER and mitochondria. In general, the cytosolic pH (pH_c_) is acidic to protect against further acidification triggered by external environmental stimuli for main two reasons. First, the electrical potential across the membrane promotes positively charged proton uptake and negatively charged HCO_3_^−^ outflow through conductive channels. Second, acid equivalents are created via several metabolic activities, including ATP synthesis in mitochondria via oxidative phosphorylation and glycolysis in the cytoplasm (Fig. 5). To prevent their accumulation, protons in the cytosol must be continuously externalized across the PM. Proton-pumping ATPases, which obtain energy from ATP, function in the PM of epithelial cells. Although this process removes protons from the cytosol, the major function of these ATPases is the maintenance of the intracellular pH.

**Fig. 6:**
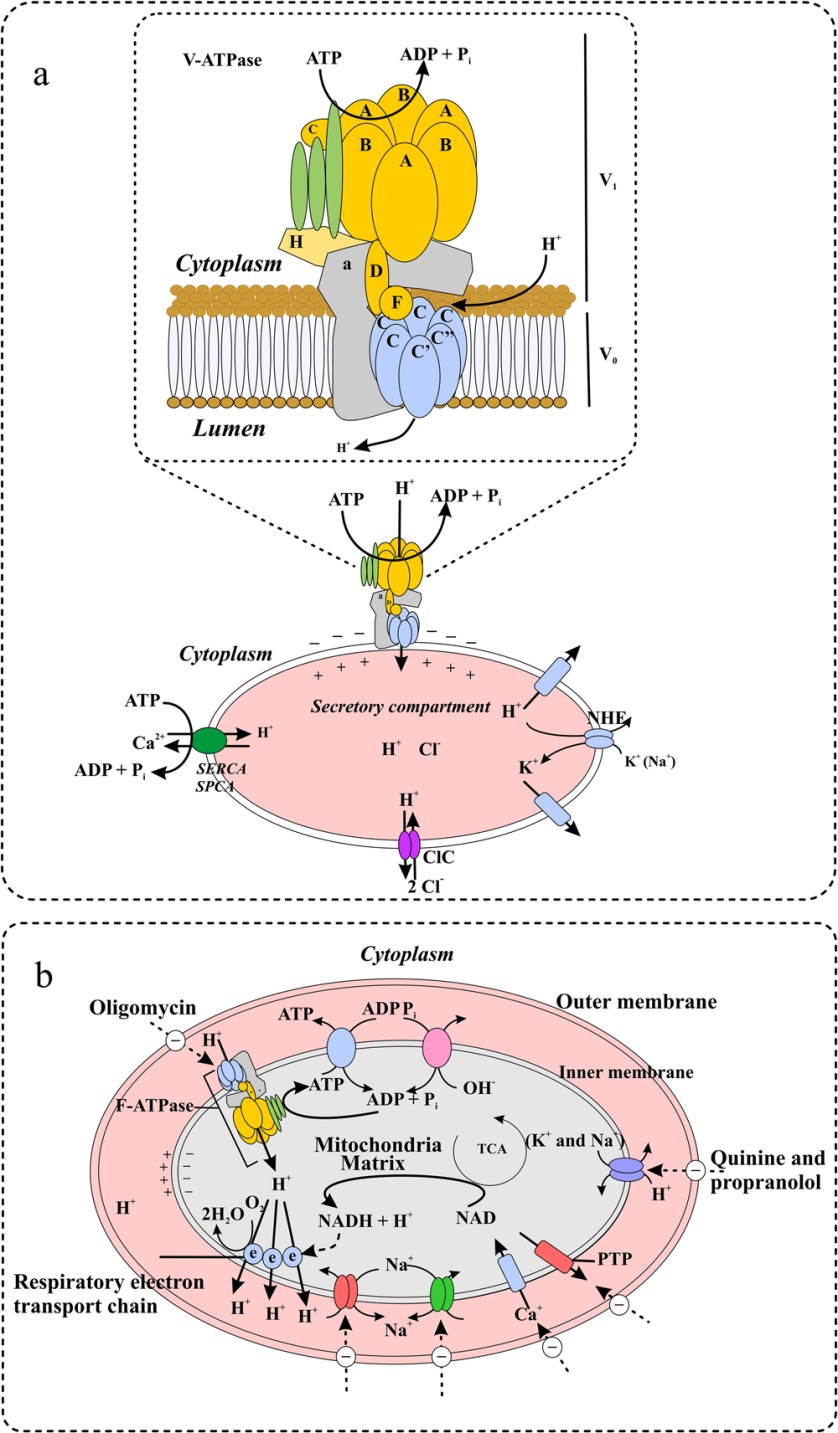
Mechanistic modification of pH in algal metabolism. **a** Regulation of pH in secretory and endocytic compartments. In endocytic and secretory pathways, acidification involves roughly balanced H^+^ efflux and influx pathways and counterions (cation and anion) of conductance. Active H^+^ accumulation in these compartments is achieved via V_1_V_0_/V-ATPases (vacuolar type H + -ATP hydrolases). These multimeric compartments comprise 14 different subunits (V_1_: A_3_, B_3_, C_1_, D_1_, E_2_, F_1_, and G_2_) and (V_0_: a_1_, d_1_, e_n_, c_4,5_, c’_1_, and c”_1_) subunits. The electrogenic exchangers (2Cl^−^/1H^+^) belong to the Cl^-^ transporter and channel in the CIC family, and make substantial contributions to optimal luminal acidification by providing anions that tend to neutralize the positive charges in the lumen, which decreases V-ATPases activity. The other H^+^ efflux pathways involve sarcoplasmic and endoplasmic reticulum Ca^2+^-ATPases (SERCAs), secretory pathway Ca^2+^-ATPases (SPCAs), the alkali cation–H^+^ (nonselective) exchangers Na^+^–H^+^ exchanger 6 (NHE6)–NHE9 and Zn^2+^-sensitive voltage-based H^+^ conductance. In addition, a cation, i.e., K^+^, which is also present in lumen of endocytic and secretory compartments, may act as a counterion via extrusion, although the carriers are ill-defined, and may contribute substantially to pH homeostasis. **b** Regulation of the pH in the mitochondrial matrix. The respiratory electron transport chain (e) in mitochondria moves H^+^ out of the matrix and into the intermembrane space, in where the F-ATPase permit the re-entry of H^+^, a process that is coupled with ATP production. Intramitochondrial pH homeostasis is established by the interplay among (i) the respiratory chain, (ii) F-ATPase and (iii) the proton transport equivalents that are directly coupled to other inorganic cations. In the matrix, Ca^2+^accumulates through a ruthenium red-sensitive Ca^2+^ channel/uniport, that is driven by the electrical potential generated by the respiratory chain. Through an exchanger in the matrix, the excessive accumulation of Ca^2+^ is prevented by Na^+^-coupled Ca^2+^ efflux. In addition, Ca^2+^ can also be eliminated from the matrix by the transient opening of the (PTP) permeability transition pore. Excessive Na^+^ taken up through the Na^+^ to Ca^2+^ exchange is, in turn, prevented by the efflux of Na^+^ in exchange for H^+^.

Via their accurate delivery to the ER, newly synthesized glycoproteins are crucial for cell development and survival^54^. However, the interaction between these cargo molecules and receptors can be influenced by the protein protonation status. The pH of the luminal membrane is not continuous throughout the secretory routes^54^, with the pH of the ER nearly neutral and that of the cytosol being slightly acidic. Proton pumping V-ATPases (vacuolar-type) comprise the principal mechanisms for delivering protons into the lumen. The multisubunit V-ATPase complexes are composed of two domains: six distinct integral membrane complex subunits (V0) and eight peripheral complex subunits (V1) (Fig. 6a). In the V_1_ complexes, chemical energy is stored as ATP for use in the mechanical force that is required for proton displacement, and V_0_ complexes translocate protons across the membrane. Under stress conditions, V-ATPases might diffuse the pH gradient throughout the whole secretory route^55^. The alternative isoform subunits of V-ATPases may explain this variation in pH.

These isoforms are often related to the unique needs of specialized tissues and organelles. They may alter the V-ATPase transport, counterion conductance, and degree of proton dissipation and leakage. The pumping rate in the final stage depends on redox state enzymes^56^. The counter ion conductance dictates the organelle that can be acidified. Even a small neutralizing conductance enables a V-ATPase pump to realize the maximal pH gradient. Two reasons account for this potential outcome. First, as the secretory system is always operational, fluid from any subcompartment is transported to the membrane and extracellular space. Because of this, the time available for luminal acidification is limited, and the degree of acidification is thus regulated by the organellar flow, H^+^ rate pumping, and counterion permeation. Second, H^+^ ions leak back into the cytosol, reducing the capacity of a pump to acidify the lumen. A steady state is reached when leakage (which increases with acidity) equals the pumping rate (which decreases with acidification). Thus, these effects are synergistic, and the secondary pathway components (e.g., secretory granules) are significantly more acidic than the initial components (e.g., the ER).

In contrast to the secretory lumen, the mitochondrial matrix is clearly alkaline due to H^+^ movement across inner membranes via electron transport chain mechanisms^57^ (Fig. 6b). The transmembrane pH gradient, in conjunction with the electrical potential created^58^ by the electrogenic proton-extrusion process, serves as a motive source of protons (ψ_H_^+^), which are used by the inner-membrane H^+^-ATPase (F_1_F_0_-ATPase) to produce ATP from ADP and inorganic phosphate. The electrochemical gradient also controls Na^+^, K^+^, and Ca^+^ homeostasis via alkali cation H^+^ exchange and Ca^2+^ pathways^59^. In turn, matrix Ca^2+^ regulates various TCA cycle dehydrogenases that are necessary for energy generation of the mitochondria^60^ F_1_F_0_-ATPase^60^ and the adenine nucleotide translocase^61^, which have been proposed to synchronize cellular needs for energy production^58^. Thus, variations in matric pH play important roles in the control of mitochondrial metabolism and other systems that rely on mitochondrial functions, such as cellular respiration. pH homeostasis regulation in mitochondria has in turn been implicated in mitochondrion-dependent apoptosis. With parallel cytosol acidification, apoptotic factors, i.e., apoptosis regulators/proteins, induce mitochondrial matrix alkalinization. This alkalinization requires F_1_F_0_-ATPase activity, which, in contrast to its canonical function as a synthase, consumes ATP during apoptosis to pump protons from the lumen into the intermembrane space and then to the cytosol^62^. In summary, acidification of the cytosol clearly increases the proteolytic activity of caspases via cytochrome C released from mitochondria. This may be one of the primary reasons, along with TS, for the modified metabolic profile of *H. akashiwo* and the related physiological changes observed in this study.

We have shown that the combination of ocean warming and acidification impacts on the *H. akashiwo* genotype and the phenotype is acquires, which are related to elemental (C:N:P) accumulation, stoichiometry changes, and macromolecular variations. We found no evidence of any cellular macromolecules or elements that have been positively influenced by climate change conditions. Our results imply that future ocean conditions may profoundly change the element ratio with concurrent metabolic shifts in the *H. akashiwo* community, specifically in a temperate environment. We also discussed the signaling process of *H. akashiwo* for the first time to best of our knowledge which causes metabolic alterations that induce physiological changes when exposed to elevated temperature and *p*CO_2_. This discovered functional response (signaling process) of *H. akashiwo* may be used as a model to study other harmful bloom species in future ocean phytoplankton biogeography. Although our results reveal insights into the signal transduction, additional studies using mutation or protein structure-function analysis related to cellular macromolecular contents are warranted to understand in detail the individual impact of temperature and pH changes in response to future ocean warming and acidifying conditions.

## Materials and methods

### Algal collection and experimental design

The raphidophyte (*H. akashiwo*) was isolated from the Qinhuangdao coastal waters, Bohai Sea (temperate environment) in 2018. Once isolated the stock culture was maintained in an artificial seawater medium (ASM) based on the Aquil recipe^63^ supplemented with 0.85% NaCl and a 2% antibiotic solution (1000 U ml^−1^ penicillin and 10,000 µg ml^−1^ streptomycin) with a light intensity of 100–120 µmol photons m^−2^ s^−1^ by white light in a 12:12 light: dark cycle at a temperature of 21 °C. Stock culture was maintained using the same conditions as in this experiment used in the present ocean conditions, that is, continuous bubbling at 400 ppm and 21 °C temperature over a year. From stock culture, cells were transferred into three 1000 mL tissue culture flasks containing ASM medium subjected to the present, 21 °C:400 ppm (Low Temperature-LT; Low Carbon-dioxide-LC) and future ocean conditions, 25 °C and 1000 ppm (High Temperature-HT; High Carbon-dioxide-HC). Experiments were carried out by two different climate-controlled incubation chambers set to the abovementioned (LTLC and HTHC) conditions. Different *p*CO_2_ conditions were created by bubbling locally purchased gas, which was added to central gas bottles through 0.22 µm filtration using silicon tubes^64^. Experimental incubations lasted for 20 generations following the protocol reported by Fu et al.^2^ under the above-mentioned condition. At each generation, cells were transferred to a new culture medium during the mid-exponential stage, where the samples were collected for physiological analysis.

To minimize changes in the carbonate chemistry caused by photosynthesis, the cell concentration was controlled at 1000–2000 cells mL^−1^. Triplicate samples were collected each day (1–3 ml aliquots) for density analysis and preserved in Lugol’s solution. Triplicate samples were analyzed immediately with an AE 2000 inverted microscope (Motic Group Co., Ltd., China) and a hemocytometer. Cell density data were used to monitor the growth rate, which was calculated as the daily exponential rate of change in density: µ (d^−1^) = in (*N*_f_/*N*_i_)/*dt*, where *N*_f_ is the cell density at a given time point for a sample, *N*_i_ is the cell density from the previous day measured at the same time of day, and *dt* is the time interval in days between these two sampling points. Triplicate samples from the mid-exponential stage of the 1st, 10th^,^ and 20th generations were collected to perform all the below mentioned physiochemical analyses. In addition, triplicate samples of 20th generation LTLC and HTHC samples were collected for transcriptome analysis.

### Analysis of seawater carbonate chemistry

Every morning at the same time, the pH was measured with a pH probe (Mettler Toledo DL 15 Titrator, Sweden), which was calibrated with National Bureau of Standard (NBS) buffer solutions of pH 7.0 and pH 10 (Sigma‒Aldrich, USA). To calculate the total alkalinity (TA), samples were passed through cellulose acetate membranes (0.45 µm pore size), treated with a saturated HgCl_2_ solution, and stored at 4 °C. The TA was analyzed by Gran acidimetric titration with a TA analyzer (AS-Alk1 + , Apollo SciTech), and 0.1 mol^−1^ HCl was used to titrate samples. To confirm the accuracy of the TA measurements, certified references were employed (±2 µmol kg^−1^). The dissolved inorganic carbon (DIC) concentration was estimated with a Shimadzu Total Organic Carbon Analyzer (TOC-5000A, Japan). CO_2_SYS software was used to convert pH_NBS_ to pH and to evaluate other parameters of the carbonate system based on pH, DIC, alkalinity, and salinity^65^. The obtained carbonate chemistry data are shown in Table S2, Supplementary material.

### Photosynthetic, elemental and macromolecular analysis

*H. akashiwo* quantum yields from LTLC and HTHC cells were determined using XE-PAM (Walz, Germany) with light saturation at 5000 µmol m^−2^ s^−1^. Cells under LTLC and HTHC conditions were filtered through polycarbonate membranes to assess CA activity using Wilbur and Anderson’s method^66^. Triplicate samples of both LTLC and HTHC cells were filtered (<16 kPa) through precombusted (treated for 4 h at 450 °C) Whatman GF/F filters (0.7 mm pore size) for determination of the carbon: nitrogen: phosphorus (C:N:P) ratio and chlorophyll-*a*, carbohydrate, and lipid level estimation. Samples of DNA, RNA, and protein were collected using a precombusted Whatman Nucleopore (25 mm pore size). The samples were flash-frozen in liquid nitrogen and stored at −20 °C until analysis.

C and N filters were dried overnight at 55 °C before being pelleted into tin capsules and analyzed with a CHNS/O analyzer (Perkin Elmer 2400 Series). Phosphate was dried and hydrolyzed (in 0.1 M HCl) at 90 °C using a technique established by Solorzano and Sharp^67^. The carbohydrate level was estimated using the TPTZ (2,4,6-Tris (2-pyridyl) -s triazine) method^68^ followed by the two-stage acid hydrolysis technique^69^ and neutralized with sodium hydroxide. To quantify chlorophyll levels, high-performance liquid chromatography (HPLC, Agilent 1100) was performed with standards obtained from DHI Water & Environment, Hørsholm, Denmark. Lipids were extracted following a method described by Folch et al. ^70^ with HPLC-grade solvents (refer Supplementary material for details). The contents of the dried lipid extracts (20–140 µg) were identified by acid-dichromate colorimetry^71^ using glyceryl tripalmitate as a standard in a spectrophotometer (Shimadzu UV-1800). To quantify the phosphorus content, a small fraction of the lipids was selected and air-dried at room temperature to remove chloroform before being analyzed, in a process similar to that used to measure the total P content in the samples. Protein was extracted by bead milling in 2% sodium dodecyl sulfate (SDS) buffer. Bead milling was conducted at 7.0 m s^−1^ three times for one minute each time, with samples stored on ice for 3 min to prevent deterioration due to heating. The amount of extracted protein extraction was then measured using a Bio–Rad DC assay kit with a microplate reader; bovine gamma (Bio–Rad) was the standard^72^. Protein extracted quickly and easily with SDS buffer, and the measurement indicated less than a 5% difference across samples.

A modified microplate technique was performed (Supplementary material) to analyze nucleic acids^73^. The RNA level was measured against an *Escherichia coli* (eukaryote) ribosomal RNA standard (Ambion #7940), while DNA was quantified based on an IX calf thymus DNA standard (Sigma #D4522)^15^. To verify the RNA content, the phenol-chloroform method was used^74^. Although both approaches produced similar findings, a method developed by Berdalet et al.^73^ produced a highly significant difference in yield (*p* < 0.05), showing high extraction efficiency (Supplementary material), and these results are reported herein. The measurements of RNA, DNA, phospholipid, and cellular P levels were considered to indicate residual P levels. Residual P accumulates intercellularly in plankton through surface absorption as orthophosphate (PO_4_) polyphosphate ([PO_4_]_2_[PO_3_]n) or orthophosphate^75, 76^. Hence, the residual P level was considered to be represented by the molecular formula for orthophosphate (PO_4_) and was measured to determine mass accumulation and for comparison with the levels of other macromolecules.

### RNA extraction and sequencing

One liter of medium with 20th generation cells from triplicate samples grown under HTHC and LTLC conditions was filtered through polycarbonate membranes (47 mm and 3 µm pore sizes). The filtrates were instantly flash-frozen and stored in liquid nitrogen. Total RNA was extracted by TRIzol reagent (Invitrogen, USA) following the manufacturer’s instructions. For each sample, ~5 µg of RNA was used to construct a cDNA library. The RNA quality was analyzed using an Agilent 2100 Bioanalyzer (Agilent Technologies, USA). Following the quality assessment, poly (A) messenger RNA was extracted from preserved RNA samples using oligo (dT)-attached magnetic beads (Illumina, CA, USA) and then fragmented into small pieces. Random hexamer primers were used to prime and transcribe the RNA fragments to cDNA. First-strand cDNA was then generated to create the final cDNA library on an Illumina platform (San Diego, USA) with 90 nucleotide pair reads. Next, RNA was isolated using a QIAquick Gel Extraction Kit (Qiagen, Netherlands). The cDNA library was sequenced using paired-end reads of 90 nucleotides on an Illumina HiSeq 2000 (San Diego, USA).

### De novo sequence assembly and functional annotation

Before further analysis, we eliminated the low-quality reads and those with a high percentage (0.05%) of unknown bases (N) using PrintSeq (v0.20.4) and Trimmomatic (v0.38)^77^. The resulting Q_20_ clean reads were then matched to reference gene sequences using Bowtie2 (v2.2.5). The Trinity de novo assembler (v2.8.4) was used to perform de novo sequence assembly based on high-quality reads. The raw assembly data were filtered using TransDecoder (v5.5.0) on the basis of a 300-nucleotide transcript length and identifiable CDSs. Furthermore, using BUSCO (v3)^78^, alternative CDS splice isoforms or transcripts were selected as unigenes. These transcripts were annotated with the NCBI-NR and NCBI-NT^79^, Swiss-Prot, GO, and KEGG^80^ databases using InterProScan (v5.33-72.0)^81^. The binominal^82^ and Poisson distribution^83^ were determined to identify DETs/DEGs with a false discovery rate (FDR) < 0.01 and a fold change >2. Using the KEGG database, metabolic pathway analysis of the DETs was performed using phyper (R software). The following *p* value formula was used for the hypergeometric test:

1 $${{{{{\rm{P}}}}}}=1-\mathop{\sum }\limits_{i=0}^{m-1}\frac{\left(\begin{array}{c}M\\ i\end{array}\right)\left(\begin{array}{c}N-M\\ n-i\end{array}\right)}{\left(\begin{array}{c}N\\ n\end{array}\right)}$$

Then, we calculated the FDR for each *p* value, and terms with an FDR <0.01 were considered to be significantly enriched.

### Statistics and reproducibility

Data from the experimental results were analyzed with Prism 6 (GraphPad Software, Inc., La Jolla, CA, USA) by two-way ANOVA, and grouped (multiple *t*-test) analysis. Significance was determined to be indicated with a *p* value < 0.05.

### Reporting summary

Further information on research design is available in the Nature Portfolio Reporting Summary linked to this article.

## Supplementary information

Supplementary Material

Description of Additional Supplementary Files

Supplementary data 1

Supplementary data 2

Supplementary data 3

Supplementary data 4

Supplementary data 5

Supplementary data 6

Supplementary data 7

Supplementary data 8

Reporting Summary

## Data Availability

The transcriptomic data generated in this study are available in Gene Expression Omnibus (accession ID: GSE223198). Source data for figure panels 1-3 can be found in supplementary data 1. Gene expression data for different metabolisms discussed are available in supplementary data 2–8.
