# Supplementary material for: Ocean warming and acidification affect the transitional C:N:P ratio and macromolecular accumulation in the harmful raphidophyte Heterosigma akashiwo
Source: Commun Biol. 2023 Feb 6;6:151. doi: 10.1038/s42003-023-04524-8 (PMC9902392; doi:10.1038/s42003-023-04524-8)
Supplement: Supplementary file 1 — Supplementary Material [file 42003_2023_4524_MOESM1_ESM.pdf]

Supplemental Material: Tables and methods

**Table S1.** Number of identified unigenes annotation in functional database		
Database	**Number of unigenes**	**Percentage %**
Total	126,555	100%
Annotated in NR	49,917	39.44%
Annotated in NT	12,872	10.17%
Annotated in Swiss-Prot	29,376	23.21%
Annotated in KEGG	34,805	27.50%
Annotated in KOG	33,901	26.79%
Annotated in Pfam	48,753	38.52%
Annotated in GO	25,169	19.89%
Annotated in intersection	3,538	2.80%
Total unigenes/ overall	64,387	50.88%

Table S2. Determination of carbonate chemistry at different generations of *H. Akashiwo* under combining LTLC (21ºC, 400 ppm) and HTHC (25ºC, 1000 ppm) conditions.

Parameters	1st generation		10th generation		20th generation	
	LTLC	HTHC	LTLC	HTHC	LTLC	HTHC
pH_{NBS}	8.19 ± 0.08	7.83 ± 0.04	8.17 ± 0.09	7.79 ± 0.08	8.17 ± 0.1	7.83 ± 0.03
DIC	1863 ± 7.4	1982 ± 0.8	1851 ± 9.2	1933 ± 1.4	1759 ± 3.2	1855 ± 0.9
HCO_3^-	2014 ± 11	2127 ± 11	2023 ± 14	2042 ± 11	2017 ± 20	2055 ± 11
CO_3^{-2}	193 ± 2.7	177 ± 6.3	186 ± 2.1	159 ± 3.3	190 ± 7.2	149 ± 3.3
CO_2	15.44 ± 3.1	22.18 ± 1.9	16.02 ± 2.2	23.13 ± 5.7	15.89 ± 1.1	22.82 ± 5.9

Supplementary Methods

Modifications to Lipid analysis

The lipid extraction protocol used was that of Folch et al.[1] modified to include the addition of ultra-pure water to freeze-dried samples before the addition of Folch extraction solvent (2:1 chloroform: methanol) to generate the expected ratios of chloroform: methanol: water in the final extraction solvent since this method assumes the sample is wet tissue. After the addition of water, samples were rapidly frozen and re-thawed twice before the addition of solvents to enhance extraction[2]. Additionally, extractions were performed four times and included 2 minutes of sonication using a sonic bath to further enhance extraction. Pooled extracts were purified by partitioning with 0.88% KCl[1], dried under a continuous stream of N_2 gas, and redissolved in 100% chloroform to provide additional purification.

Description of DNA/RNA Methods

The method used in this study by Berdalet et al.[3] for the extraction of RNA and DNA involves a detergent-based extraction buffer. Sub-samples of each extract were diluted, treated with RNase, DNase, or RNase+DNase, and stained with SYBR Green II. DNA and RNA were then determined by SYBR Green II fluorescence. Samples in the filter were extracted by bead milling in the final extraction buffer (0.5% n-lauryl sarcosine, 5mM Tris, 1mM EDTA) four times per 30 seconds at 6.5 m s^{-1}, and between each time samples were placed on ice for 2 minutes. Samples were then kept at room temperature for 40-50 min during which they were vortexed at high speed for 25-30 seconds every 10 minutes. The assay was scaled to a microplate format and measurement of RNA and DNA fluorescence was performed in a 96-well opaque black microplate using a microplate reader (SpectraMax M3, Molecular Devices). In addition, RNA analyses were performed on HTHC and LTLC cells using Trizol (ThermoFisher Scientific) to reduce the loss of RNA during isolation and cleaning steps and account for consistent losses of RNA during solvent partitioning. Samples on polycarbonate filters were extracted in 1ml Trizol solution and immediate bead milling four times for 25-30 seconds at 6.5 m s^{-1}, with samples on ice for 2 minutes between each round of bead milling. After partitioning of the Trizol extract with chloroform and centrifugation, ~75% of the RNA-containing upper aqueous phase of the extract was removed to avoid the collection of the DNA-containing interphase. An aliquot of pure Trizol-chloroform aqueous upper phase was then added to the remaining Trizol-chloroform extract. The pure Trizol-chloroform aqueous upper phase added to the extracts was produced earlier by making a 5:1 mixture of pure Trizol reagent and chloroform, allowing this to partition overnight, then removing and storing this pure upper aqueous phase at -20°C. The Trizol-chloroform extract was then mixed for 30 seconds by vortexing, centrifuged, and ~75% of the resulting RNA-containing upper aqueous phase was removed and pooled with the previously removed upper phase. This partitioning process was repeated and served to remove more RNA from the upper aqueous phase while limiting the removal of contaminating DNA from the interphase while also capturing more RNA that may have been lost to the phenol-chloroform phase during initial partitioning.

The combined aqueous extracts were then partitioned again with chloroform to remove the remaining phenol contamination. The RNA was then precipitated from aqueous extract with

isopropanol, with 100 µg RNA-grade Glycogen added as a co-precipitant. RNA was allowed to precipitate for 48 hours at -20°C. The resulting RNA pellet received two washes with 75% ethanol to remove any residual phenol and salts. When re-dissolving the RNA pellet in RNase-free water, the additional steps of freezing the extract at -80°C after the addition of water and then warming it to 60°C for 5 min were added to enhance+2Q J e RNA dissolution. In parallel with the sample RNA extractions and analyses, triplicate spiked blank samples containing clean polycarbonate filters and 7 µg RNA standard (*E. coli* ribosomal RNA, Ambion #7940) were also processed as described above. This was done to account for the consistent fraction of RNA lost during the various isolation and purification steps (~35% lost based on spike recovery) and the extraction efficiency of the spiked blank samples was used as a correction factor for the sample RNA measurements. This modified phenol-chloroform extraction and the method of Berdalet et al.[3] provided similar results for *H. akashiwo* at both growth conditions Additional samples were processed only with the Berdalet et al. [3] method due to its greater ease, consistency, apparent extraction efficiency, and simultaneous quantification of DNA.

References

1 Folch, J., Lees, M. & Sloane Stanley, G. H. simple method for the isolation and purification of total lipids from animal tissues. *J biol Chem* **226**, 497-509 (1957).

2 Ryckebosch, E., Bruneel, C., Muylaert, K. & Foubert, L. T. Microalgae as an alternative source of omega-3 long chain polyunsaturated fatty acids. **24**, 128-130 (2012).

3 Berdalet, E., Roldán, C., Olivar, M. P. & Lysnes, K. Quantifying RNA and DNA in planktonic organisms with SYBR Green II and nucleases. Part A. Optimisation of the assay. *Scientia Marina* **69**, 1-16 (2005).
