# Supplementary material for: Ocean warming and acidification affect the transitional C:N:P ratio and macromolecular accumulation in the harmful raphidophyte Heterosigma akashiwo
Source: Commun Biol. 2023 Feb 6;6:151. doi: 10.1038/s42003-023-04524-8 (PMC9902392; doi:10.1038/s42003-023-04524-8)
Supplement: Supplementary file 2 — Description of Additional Supplementary Files [file 42003_2023_4524_MOESM2_ESM.pdf]

Description of Additional Supplementary Files

File name: Supplementary Data 1

Description: The source data behind the graphs in the paper.

File name: Supplementary Data 2

Description: : Overall DEGs identified in this study.

File name: Supplementary Data 3

Description: DEGs involved in energy metabolism.

File name: Supplementary Data 4

Description: DEGs involved in carbon acquisition and utilization.

File name: Supplementary Data 5

Description: DEGs involved in lipid biosynthesis and degradation.

File name: Supplementary Data 6

Description: DEGs involved in DNA functional process.

File name: Supplementary Data 7

Description: DEGs involved in RNA functional process.

File name: Supplementary Data 8

Description: DEGs involved in protein formation and degradation.
